# Commentary: A Host-Produced Quorum-Sensing Autoinducer Controls a Phage Lysis-Lysogeny Decision

**DOI:** 10.3389/fmicb.2019.01201

**Published:** 2019-06-03

**Authors:** Xiaolong Liang, Mark Radosevich

**Affiliations:** Department of Biosystems Engineering and Soil Science, The University of Tennessee, Knoxville, TN, United States

**Keywords:** lysis, lysogeny, quorum sensing (QS), phage, host, communication, interaction

Phage reproduction depends on bacterial host cells, and thus it is critical for temperate phages to modulate their reproduction strategy in response to host cell densities. Silpe and Bassler (2019) elucidate the regulatory mechanism controlling host cell density-dependent lysis-lysogeny decision made by Vibrio phage that is dependent upon a host quorum-sensing system.

Phages, either obligate lytic or temperate, are viruses that infect bacteria. Within the intracellular stage of infection, a temperate phage can either enter the lytic cycle releasing progeny virions upon lysis of the host cell or lysogenize the host cell by stably integrating its genome within the host genome as a prophage. Upon certain environmental cues, a prophage of a lysogenized cell may become induced and enter the lytic reproductive cycle. The lysis-lysogeny decision may have significant influence on host metabolism, population dynamics, ecological processes, and phage dissemination (Díaz-Muñoz et al., 2017). Host cell density has been proposed in previous studies to impact phage lysis-lysogeny decisions suggesting temperate phages may have specific mechanisms to sense host cell density (Ghosh et al., 2009; Ofir and Sorek, 2018). The first experimental evidence of cell-density dependent prophage induction linked to quorum-sensing systems was demonstrated by Ghosh et al. (2009) in soil and groundwater bacteria and a model *E. coli* system. A model bacterial system was used to reveal the basis of the molecular regulatory mechanism (Ghosh et al., 2009). The homoserine lactone-based induction mechanism was SOS (i.e., *recA*) independent. Many studies have also shown that phage abundance is positively correlated with host density in a variety of environments (Brum et al., 2016; Liang et al., 2019), and lytic infections are favored under favorable conditions supporting rapid cell growth, whereas lysogeny becomes more common under conditions less favorable for growth with lower cell density (Breitbart et al., 2018). However, in some high host-density environments, such as the animal gut, lysogenic replication may be favored following the Piggyback-The-Winner model (Silveira and Rohwer, 2016). It is critical to investigate the molecular mechanisms behind the phage-host interactions for better understanding of microbial ecology and processes.

Bacteria can produce, secrete, and detect signal molecules (“autoinducer,” AI) for cell-cell communication to coordinate a wide range of group behaviors; a process called quorum sensing (QS) that is cell density dependent. Papenfort et al. (2017) recently characterized a new QS circuit that comprises a cytoplasmic receptor and transcription factor, VqmA, and an AI 3,5-dimethylpyrazin-2-ol (DPO). The authors hypothesized that phages might be capable of utilizing the host QS system for lysis-lysogeny decisions, thus Silpe and Bassler (2019) took a further step and evaluated the hypothesis. Silpe and Bassler (2019) collected VqmA homologs to identify DPO-binding proteins of viral origin through bioinformatics analyses. Interestingly, one such protein, VqmA_phage_ of vibriophage VP882, can cause host cell lysis and cell density decline, in a similar way as mitomycin C (MMC) inducing lytic reproduction of VP882 phages. Silpe and Bassler (2019) demonstrated that the activation of VqmA_phage_ by binding to host-produced QS AI launches the phage lytic life cycle (Figure 1). This gives a new perspective on phage-host interaction in which phage proteins use host-signaling molecules as cues for reproductive fate (i.e., lytic-lysogeny) decisions.

**Figure 1:**
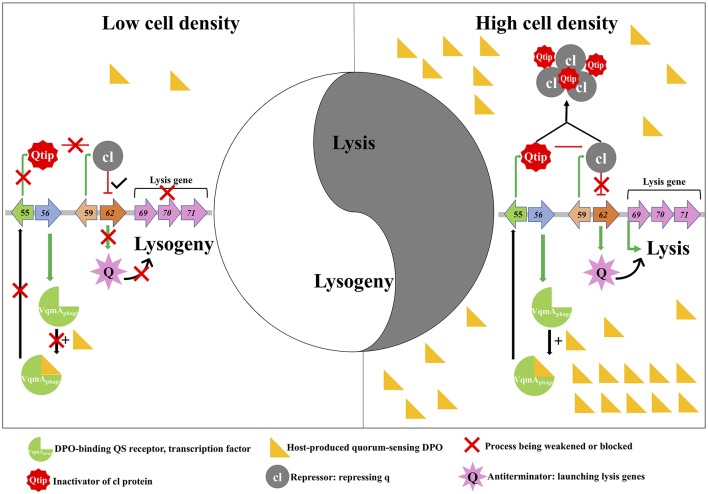
Quorum sensing-mediated lysis-lysogeny decisions of phage VP882. The genes of phage VP882 are characterized in the repressed **(Left Side)** and induced **(Right Side)** pathways. gp56 encodes the VqmA_phage_ protein that can bind the host quorum-sensing autoinducer (DPO). The Q anti-terminator can activate lysis genes gp69-71 inducing lysis **(Right)**, whereas cl represses q thereby promoting lysogeny **(Left)**. At high cell density, high levels of host-produced DPO promotes Q-tip anti-repressor production. Q-tip then sequesters the cl repressor that allows Q to launch the lysis pathway. However, cl repressor can function at low cell density leading to lysogeny.

Silpe and Bassler (2019) went on to demonstrate that phage gene gp62 is required for lysis, and the gp62-coded protein Q directly targets the lysis genes gp69-71 triggering the lytic cycle of VP882 prophages. Meanwhile, the authors also examined the regulation of lysogeny of VP882 and discovered that gp59-coded cl repressor can directly repress the gene gp62 promoting lysogeny. The authors then proved that MMC-induced lytic program of VP882 phages is based on host SOS response-dependent cleavage of the cl-repressor. In efforts to connect the phage regulatory systems of MMC-induced lysis and DPO-driven VqmA_phage_-mediated lysis, the authors showed that Q production occurs first and thereafter Q activates gp69 launching the lysis program. However, further investigation revealed that the pathway of VqmA_phage_-mediated lysis is distinct from that of MMC-induced phage lysis. The DPO-activated phage-produced VqmA_phage_ binds upstream of phage gene gp55 activating its expression that leads to the anti-repressor Q-tip production. The function of the 79-amino acid protein Q-tip is to directly aggregate the cl repressor and prevent cl from binding to the promoter of q which allows Q production inducing lysis (Figure 1). Specifically, DPO is the determinant driver of phage lytic cycles, which enables the connection of lysis-lysogeny decision with host cell density-dependent QS.

QS is also involved in other phage-bacteria interactions. Patterson et al. (2016) discovered that high *Serratia* cell density can promote CRISPR-Cas immune defense through acyl-homoserine lactone-based communication, whereas many phages also encode CRISPR-Cas inhibitor proteins to fight back (Rauch et al., 2017). Plenty of studies have inferred the association of lysis-lysogeny decisions of phage communities with bacterial host density, and these reports are significant for revealing one potential molecular mechanism behind it. It is important for phages inside host cells to have evolved mechanisms (e.g., QS-mediated) to sense their host density and make wise coordinated decisions on lysis-lysogeny such that phages can maximize propagation and avoid depleting susceptible hosts by estimating the successful infection rates for offspring phages based on host cell density (Díaz-Muñoz et al., 2017).

*Bacillus* phages within the *subtilis* phage beta group can also sense the population level of nearby relatives and strongly influence the lysis-lysogeny decisions through phage-encoded peptides (Erez et al., 2017). The communication peptide employed is phage-specific, which means phages produce different peptides and only influence members of their own kind. The arbitrium system as it is known thus enables an infecting phage to determine the extent of previous infections that are in an active lytic mode of replication. During lytic replication the phage-encoded six amino-acid-long signaling peptide is produced by infected bacteria and if the signal reaches a sufficiently high concentration the phage in subsequent infections will enter the lysogenic mode. This is quite different from the *Vibrio* phage system report by Silpe and Bassler (2019) in which the lytic/lysogenic decision responds to the host bacteria-encoded and produced communication signal.

In addressing the generality of vqmA_phage_-Q-tip-like lysis-lysogeny regulation pathway, Silpe and Bassler (2019) found that Q-tip-type proteins are conserved and function only among related phages. Small genes and ORFs with predicted DNA-binding domains in the same locations of vqmA_phage_-Q-tip were identified in four phage genomes, and none of the above DNA binding proteins were VqmA_phage_ homologs leading the authors to speculate that these phage-originated proteins respond to different host-produced signals to initiate transcription of anti-repressor-encoding genes. Intrigued by these discoveries, we propose a complementary hypothesis targeted at the microbial ecosystem level. Because quorum-sensing signals act in a rather taxon-specific manner, we hypothesize that any single quorum-sensing signal should only induce prophages within a small subset of closely-related host bacteria within vastly more diverse microbial communities.

## Author Contributions

XL proposed the idea and drafted the article. MR helped XL develop the idea and improve the article.

### Conflict of Interest Statement

The authors declare that the research was conducted in the absence of any commercial or financial relationships that could be construed as a potential conflict of interest.
